# Editorial: Interfacial strategies to manipulate tissue interactions for wound healing

**DOI:** 10.3389/fbioe.2022.995721

**Published:** 2022-08-24

**Authors:** Yazhong Bu, Guoming Sun, Da Huang, Fei Yang

**Affiliations:** ^1^ Department of Biophysics, School of Basic Medical Sciences, Health Science Center, Institute of Medical Engineering, Xi’an Jiaotong University, Xi’an, China; ^2^ Affiliated Hospital of Hebei University, Key Laboratory of Bone Metabolism and Physiology in Chronic Kidney Disease of Hebei Province College of Chemistry and Environmental Science, Institute of Life Science and Green Development, Hebei University, Baoding, China; ^3^ Linda and Bipin Doshi Department of Chemical and Biochemical Engineering, Missouri University of Science and Technology, Rolla, MO, United States; ^4^ College of Biological Science and Engineering, Fuzhou University, Fuzhou, Fujian, China; ^5^ Beijing National Laboratory for Molecular Sciences, Institute of Chemistry, Chinese Academy of Sciences, Beijing, China; ^6^ School of Chemical Sciences, University of Chinese Academy of Sciences, Beijing, China

**Keywords:** bioadhesive, wound dressing, tissue adhesion, chronic wound, interfacial strategy

Wounds are injuries that break the skin or other body tissues, which can be divided into acute and chronic ones. Due to the aged population, and increasing rates of diabetes, accidents and disasters, the number of people affected by wounds are increasing (Gwak and Sohn, 2017; Ashtikar and Wacker, 2018). The global market for wound care is expected to reach $22 billion by 2022, with a compound annual growth rate of 3.7% from 2016 to 2022 (Han and Ceilley, 2017). There are different strategies to develop wound care products and this topic focuses on those research which develops wound care products through manipulating interactions between tissues and materials.

In wound care, bioadhesives have superiority in clinical applications as tissue adhesives, hemostats, and tissue sealants. They interact with tissues through mechanical interlocking, electrostatic bonding, intermolecular bonding, and chain entanglement (Mehdizadeh and Yang, 2013). In “*Applications of Bioadhesives: A Mini Review*”, Duan et al. summarized the applications of the bioadhesives in wound healing. The first one is for non-invasive wound closure to replace the traditional invasive wound closure methods. The second is for sealing leakage, including various liquid and gas leakage. The third is immobilization for functional wound dressing or other medical device fixations. This article is expected to help researchers better understand the applications of bioadhesives and lead to related innovations.

The first stage of wound healing is hemostasis. The rapid and effective hemostasis is of great importance to improve the quality of wound healing and lifesaving (Bai et al., 2022). In “*Hydrogel-Based Biomaterials Engineered from Natural-Derived Polysaccharides and Proteins for Hemostasis and Wound Healing*”, Cheng et al. summarized the hydrogel-based biomaterials, engineered from natural polysaccharides and proteins, for hemostasis. They focused on the preparation strategies, physicochemical properties, hemostatic and wound-healing abilities of those novel biomaterials. They also highlighted the challenges that need to be addressed for the commercialization. In the future, wet surface adhesion, pressure-resistant intensity, high mechanical strength, and rapid gelatin are key features for ideal wound care materials. Besides, the biocompatibility of the synthetic procedure should also be considered.

After hemostasis, the wound goes through inflammation, proliferation, and remodeling stages before healing. The chronic wound is unable to heal normally, and it causes a significant burden to both the patient and the medical system (Han and Ceilley, 2017). In “*Biomimetic Hydrogels to Promote Wound Healing*”, Fan et al. discussed the effects of various types of hydrogels on wound pathophysiology, as well as the ideal characteristics of hydrogels for wound healing. These include biocompatibility, degradability, removability, tissue adhesiveness, and so on. Considering that wounds have different conditions, tailor-made therapies have been explored and will become increasingly relevant in the coming days.

Another major complication impairing wound healing is microbial infection. Sporotrichosis is a subcutaneous mycotic infection, which causes localized cutaneous and even severe systemic disseminated infections, and its global incidence increases every year (Etchecopaz et al., 2021), while the efficacy of current therapies is far from enough. In “*Antifungal Activity of ToAP2D Peptide Against Sporothrix globosa*”, Yan et al. synthesized antimicrobial peptides ToAP2A, ToAP2C, and ToAP2D based on the ToAP2. Compared with the template, the modified peptide had significantly better stability and enhanced the protein binding ability. They found that ToAP2D showed strongest anti-fungal activity with similar efficiency as that of itraconazole.

Skin is the largest organ in the body, playing a vital role in maintaining body functions and protecting the internal organs from hostile exterior environments (Chambers and Vukmanovic-Stejic, 2020). However, skin is easily got injured and according to World Health Organization (WHO), more than 11 million cutaneous wounds need medical care every year (Monavarian et al., 2019). In “*Recent Advances in Bioengineered Scaffolds for Cutaneous Wound Healing*”, Qin et al. highlighted the bioengineered scaffolds for skin generation. These scaffolds not only act as isolation for wounds and surroundings, but also interact with tissues to regulate cellular functions, facilitate the exchange of nutrients and wastes, or/and load cells and biomolecules for therapy.

This topic presented different strategies to promote wound healing by manipulating the interactions. These mainly include bioadhesion between tissues and materials for high stability and therapeutic efficacy. We hope that this topic will provide some insights for developing wound care materials through tissue interactions between materials and tissues.
